# Impact of fertilization depth on sunflower yield and nitrogen utilization: a perspective on soil nutrient and root system compatibility

**DOI:** 10.3389/fpls.2024.1440859

**Published:** 2024-08-14

**Authors:** Wenhao Ren, Xianyue Li, Tingxi Liu, Ning Chen, Maoxin Xin, Bin Liu, Qian Qi, Gendong Li

**Affiliations:** ^1^ College of Water Conservancy and Civil Engineering, Inner Mongolia Agricultural University, Hohhot, China; ^2^ Collaborative Innovation Center for Integrated Management of Water Resources and Water Environment in the Inner Mongolia Reaches of the Yellow River, Hohhot, China; ^3^ Research and Development of Efficient Water-saving Technology and Equipment and Research Engineering Center of Soil and Water Environment Effect in Arid Area of Inner Mongolia Autonomous Region, Hohhot, China; ^4^ Inner Mongolia Hetao Irrigation District Water Conservancy Development Center, Bayannur, China

**Keywords:** fertilization depth, root growth, nitrogen fertilizer efficiency, sunflower yield, nutrient matching between root and soil

## Abstract

**Introduction:**

The depth of fertilizer application significantly influences soil nitrate concentration (SNC), sunflower root length density (RLD), sunflower nitrogen uptake (SNU), and yield. However, current studies cannot precisely capture subtle nutrient variations between soil layers and their complex relationships with root growth. They also struggle to assess the impact of different fertilizer application depths on sunflower root development and distribution as well as their response to the spatial and temporal distribution of nutrients.

**Methods:**

The Agricultural Production Systems sIMulator (APSIM) model was employed to explore the spatial and temporal patterns of nitrogen distribution in the soil at three controlled-release fertilizer (CRF) placement depths: 5, 15, and 25 cm. This study investigated the characteristics of the root system regarding nitrogen absorption and utilization and analyzed their correlation with sunflower yield formation. Furthermore, this study introduced the modified Jaccard index (considering the compatibility between soil nitrate and root length density) to analyze soil-root interactions, providing a deeper insight into how changes in CRF placement depth affect crop growth and nitrogen uptake efficiency.

**Results:**

The results indicated that a fertilization depth of 15 cm improved the modified Jaccard index by 6.60% and 7.34% compared to 5 cm and 25 cm depths, respectively, maximizing sunflower yield (an increase of 9.44%) and nitrogen absorption rate (an increase of 5.40%). This depth promoted a greater Root Length Density (RLD), with an increases of 11.95% and 16.42% compared those at 5 cm and 25 cm, respectively, enhancing deeper root growth and improving nitrogen uptake. In contrast, shallow fertilization led to higher nitrate concentrations in the topsoil, whereas deeper fertilization increased the nitrate concentrations in the deeper soil layers.

**Discussion:**

These results provide valuable insights for precision agriculture and sustainable soil management, highlighting the importance of optimizing root nitrogen absorption through tailored fertilization strategies to enhance crop production efficiency and minimize environmental impact.

## 1 Introduction

Nitrogen is crucial for optimal crop yields and is a key nutrient for plant growth (Tabuchi et al., 2007; Heuer et al., 2017). The global utilization rate of nitrogen fertilizers is generally low, often around 40%–53% (Chen et al., 2021b; Wang et al., 2023a), leading to inefficiencies in agricultural practices, where farmers frequently apply excessive nitrogen fertilizer, surpassing the crops’ actual growth requirements (Nie et al., 2022; Wang et al., 2022a). Over-application of nitrogen fertilizers increases agricultural costs and causes environmental issues, as reported globally (Chen and Graedel, 2016; Ma et al., 2019; Chen et al., 2021a). Additionally, the excessive application of nitrogen fertilizers can contribute to increased greenhouse gas emissions, such as nitrous oxide (N_2_O), which has high global warming potential (Kong et al., 2021; Ren et al., 2021; Wu et al., 2023). Therefore, improving the efficiency of nitrogen fertilizer use is vital for global food security, sustainable economic development, and mitigating the ecological impact of agriculture. Studies have demonstrated that adopting more precise fertilization methods and improved agronomic techniques can significantly enhance nitrogen utilization efficiency (NUE) (Radočaj et al., 2022; Ren et al., 2022; Luo et al., 2023). For example, adjusting fertilization strategies, such as altering the depth of fertilization, can increase nitrogen use efficiency by 38.37%, yield by 13.83%, and reduce nitrogen loss by 70.23% compared with traditional methods (Wu et al., 2021). Changing the timing of fertilization can reduce total nitrogen input by 15% without yield loss (Xie et al., 2007). Controlled-release fertilizers (CRF), a novel type of fertilizer (Haydar et al., 2024), regulate the nitrogen release rate through coating technologies (Sim et al., 2021) and matching crop nitrogen demand (Slafer and Savin, 2018; Vejan et al., 2021). This enhances the nitrogen uptake (Li et al., 2020), lowers soil inorganic nitrogen concentration, and reduces the nitrogen loss (Azeem et al., 2014; Momesso et al., 2022; Zhang et al., 2024). Moreover, strategies to enhance crop NUE include selecting crop varieties that utilize soil nitrogen effectively and reduce nitrogen loss (Ciampitti et al., 2022). This is crucial to sustainable agricultural development. Therefore, adopting a comprehensive approach to nitrogen fertilization management and crop selection is important for improving NUE (Kant et al., 2011; Guo et al., 2020; Geng et al., 2021). Integrating these methods can effectively enhance agricultural production efficiency and reduce environmental impacts, contributing to global food security and sustainable development.

Modern agricultural research has emphasized the importance of matching plant root systems with available soil resources for efficient nutrient absorption (Jin et al., 2017; Gebre and Earl, 2021; Chen et al., 2022c). Root-foraging processes exhibit significant spatiotemporal heterogeneity owing to varying soil resource availability across different layers (Mou et al., 2012; Postma et al., 2014; Hui et al., 2022). Adjusting fertilization depth is a critical agronomic practice that can significantly affect root growth and distribution and optimize nutrient absorption efficiency, especially for nitrogen (Su et al., 2015; Chen et al., 2022a, 2024). Studies have demonstrated that spatially adjusting soil nitrate content under different rainfall conditions enhances deep soil root characteristics, indirectly improving the nitrogen nutrient status of wheat plants (Wang et al., 2022b). In trials with spring maize, increasing fertilization depth compared to conventional methods resulted in significant improvements in root length density: 18% for vertical roots, 14% for inter-row roots, and 24% for intra-row roots at soil depths of 0–1.0m. Similarly, the root surface area density increased by 39%, 17%, and 22%, respectively (Wu et al., 2022b). The application of fertilizer at depths of 15 and 25 cm, rather than 5 cm, increased maize nitrogen absorption by 8.07% and 17.41%, NUE by 17.79% and 38.37%, and maize yield by 5.68% and 13.83%, respectively (Wu et al., 2021). Optimizing the fertilization depth is crucial for improving NUE and regulating crop growth and yield (Jia et al., 2023; Wang et al., 2023b). These practices enable the agricultural production to mitigate environmental impacts while ensuring sufficient crop nutrition and promoting sustainability. This approach is particularly relevant for deep-rooting crops such as sunflowers, maize, and wheat, which can access nutrients from deeper soil layers (Connor and Hall, 1997; Wasson et al., 2012; Lynch, 2013; Zhang et al., 2023).

Sunflower, known for its strong root system adaptability, significantly enhances nitrogen absorption rate, utilization, and crop yield through deep root growth (Kiniry et al., 1992; Ma et al., 2021). Its root system can extend into deeper soil layers, effectively utilizing nutrients beyond the surface layer (Thorup-Kristensen et al., 2020). However, current agricultural research lacks sufficient analysis of the impact of moderately deep nitrogen fertilization on root distribution and spatiotemporal matching of soil nutrients in sunflower cultivation. In agricultural studies, an accurate understanding of the correlation between soil nitrate content and root length density is crucial to elucidate the interactions between crop roots and soil nutrients (Li et al., 2018; Lopez et al., 2022). This analysis deepens our understanding of how roots adapt to nutrient distribution, thereby affecting growth and yield. Despite their significance, current analytical methods often fail to precisely capture subtle differences between soil layers and their complex relationship with root growth. Therefore, the aim of this study was to develop a refined and comprehensive analytical method to accurately assess the correlation between soil nitrate distribution and root growth. We introduced an improved weighted Jaccard index (Real and Vargas, 1996; Bag et al., 2019), which considers not only the direct match between soil nitrate content and root length density but also the interrelationships among different soil layers, providing a more comprehensive analytical perspective. This method allowed us to reveal the complex interactions between crop roots and soil nutrients more precisely, providing theoretical support for optimizing fertilization strategies and improving crop production efficiency (Duan et al., 2019; Velasco-Muñoz et al., 2021).

The main objectives of this study were to (1) analyze the impact of deep CRF on the growth distribution of the sunflower root system and its role in yield and nutrient use efficiency, (2) investigate the compatibility between deep CRF and root system growth, and (3) determine the ideal CRF depth to promote an adaptive root system structure in sunflowers through more effective fertilization methods, thereby achieving higher yields. Moreover, this experiment can also contribute to refining existing soil and crop models to simulate root growth, crop yield, and nutrient use efficiency.

## 2 Materials and methods

### 2.1 Experimental site parameters

The experiment was conducted in the Ganzhaomiao Town experimental field, Linhe District, Bayannaoer City, Inner Mongolia, to investigate the effects of different fertilizer depths over two consecutive growing seasons in 2020 and 2021. The site coordinates are 107°16′42″E and 40°47′54″N, with predominantly silty loam soil (USDA classification). The 0–100 cm soil layer had an average bulk density of 1.41 g/cm^3^, an average organic matter mass ratio of 6.19 g/kg, an average hydrolysable nitrogen mass ratio of 34.43 mg/kg, an average available phosphorus mass ratio of 1.84 mg/kg, and an average available potassium mass ratio of 113.04 mg/kg. The soil salt content prior to planting averaged 3.2 g/kg, with an average pH of 8.4. The experimental site experiences an annual average temperature of 6.8 °C, precipitation of 138.8 mm, and total annual sunshine hours of 3229.9 h.

### 2.2 Experimental design and field practices

The test field comprised 12 plots, each 144 m^2^ (14 m×6 m), arranged in triplicate for the four treatments. This study used CRF with a nitrogen application rate of 225 kg/ha. Three depths of fertilizer application were tested: 5 ± 0.5 cm (low), 15 ± 0.5 cm (medium), and 25 ± 0.5 cm (high). Additionally, treatments with TNF at 225 kg/ha and a depth of 5 ± 0.5 cm were included. Plastic films were used between the plots to isolate them and to prevent water, salt, and nitrogen interactions. The CRF was the sixth generation from Luyang, with an N:P:K ratio of 28:12:10. The CRF was applied to the farmland at the corresponding depth through manual trenching before mulching. For the TNF, diammonium phosphate (containing 18% N and 46% P_2_O_5_) was used as the base fertilizer and was applied at the same depth through manual trenching before mulching, similar to the CRF treatment; topdressing consisted of urea, which was manually spread before irrigation at the bud stage. Diammonium phosphate (1/3 N) was applied before sowing, and urea (2/3 N) was manually applied before irrigation at the budding stage. Sunflowers were irrigated to a depth of 120 mm during their growth period in both 2020 and 2021 using furrow irrigation on July 14th. All the other field management practices were consistent. Sunflower (Xinjiang Sanrui, SH361) was planted using a mechanical film covering and manual planting. The planting pattern involved one film for every two rows, with sunflower plant and row spacing set at 0.4 m and 0.9 m, respectively, resulting in a planting density of approximately 27,777 plants per hectare. Sowing occurred on May 22, 2020, and May 30, 2021, with harvest dates set for September 24 and September 29 of the respective years, resulting in growth periods of 126 and 123 d.

### 2.3 Observations and measurement methods

**Figure 1:**
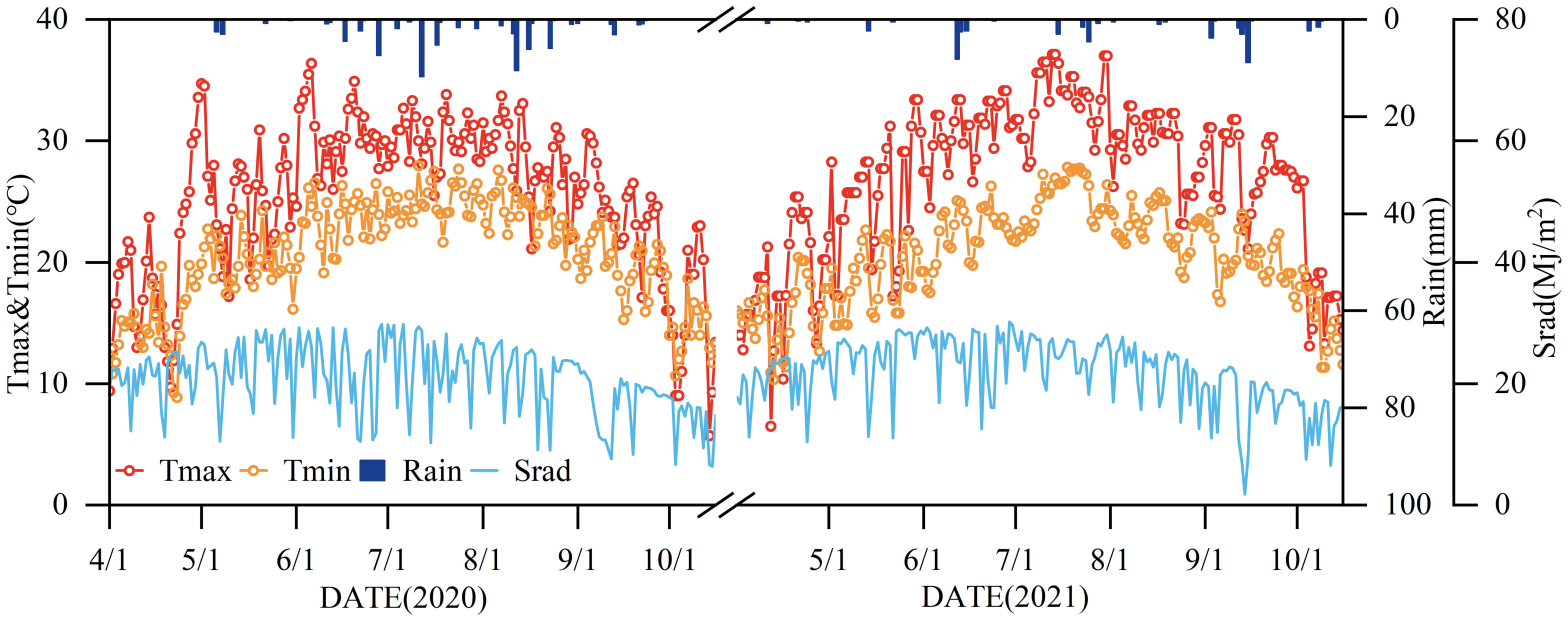
Precipitation (Rain), maximum and minimum temperatures (Tmax and Tmin), and solar radiation (Srad) during the crop fertility period in 2020 and 2021.

Meteorological data (**Figure 1**), including solar radiation, air temperature, relative humidity, precipitation, and wind speed, were collected at one-hour intervals using an automated meteorological station (Onset Computer Inc., U30, Hobo, USA) located in the experimental field.

Soil water and NO_3_-N contents were measured using a soil auger (Beijing New Landmark Soil Equipment Co., Ltd., 0301, XDB, CHN) in covered and exposed areas at vertical depths of 0–10 cm, 10–20 cm, 20–30 cm, 30–40 cm, 40–50 cm, 50–60 cm, 60–80 cm, and 80–100 cm. Samples were collected every 10–15 d, and each sample was replicated three times. The soil samples were divided into two parts: one for measuring soil moisture content and the other air-dried, crushed, and sieved through a 1 mm mesh. Subsequently, 5 g of the sample was soaked in 25 mL of 2 mol/L potassium chloride solution. After stirring and filtering, the NO_3_-N concentration was determined using an ultraviolet spectrophotometer (Beijing General Instruments Co., Ltd., TU-1901, Beijing, China).

Total nitrogen concentration in sunflowers was determined using the micro-Kjeldahl method. Every 15–20 d, five sunflower plants were randomly selected from the diagonal and central areas of each plot using a five-point method. The plant samples were ground, sieved, and digested with a H_2_SO_4_-H_2_O_2_ solution. Total nitrogen concentration was measured using a Kjeldahl apparatus (China Ocean Energy Future Technology Group, K9860, China).

During the seedling stage (June 26, 2020 and July 2, 2021, respectively), bud stage (July 20, 2020 and July 26, 2021, respectively), flowering stage (August 9, 2020 and August 12, 2021, respectively), and post-flowering stage (September 10, 2020 and September 18, 2021, respectively), root samples from well-grown sunflowers were collected from the soil cross-section at depths of 0–10 cm, 10–20 cm, 20–30 cm, 30–40 cm, 40–50 cm, 50–60 cm, 60–70 cm, 70–80 cm, and 80–100 cm. The collected root samples were washed, sun-dried, and scanned using the Epson Perfection V700 PHOTO scanner. The root parameters were determined using WinRHIZO software.

From each plot, ten sunflowers were consistently selected for measuring mature sunflower yield, and this process was repeated three times.

### 2.4 APSIM simulation building

#### 2.4.1 Introduction to the APSIM platform

The Agricultural Production Systems sIMulator (APSIM) is a versatile process-based modeling platform designed specifically for agricultural systems. It integrates multiple modules that cover various crop types, soil dynamics, and management strategies. APSIM can simulate crop growth, soil moisture, and nitrogen dynamics on a daily scale and is applicable to diverse management practices, farming systems, and environmental conditions. This model has been widely used in numerous studies to simulate agricultural production systems worldwide. The inputs to the model included daily meteorological data (such as solar radiation, maximum/minimum temperatures, and precipitation) and soil hydraulic parameters (such as bulk density, saturated water content, field capacity, and wilting point).

#### 2.4.2 Application of APSIM modules

This study adopted the APSIM simulation, integrating the modules for fertilizers, soil moisture, soil nitrogen, and sunflowers. APSIM modules that control water and soil nitrogen are particularly crucial. The APSIM SoilWat module employs a cascade water balance model to estimate water and solute movement between soil layers, surface runoff and evaporation, and drainage from the system. This module balances the water content in each soil layer based on inputs and outputs. Soil water movement occurred through saturated, unsaturated, and oversaturated flows, each of which had specific model equations and parameters. The SoilN module manages the plant-available nitrogen supply, nitrate leaching, and nitrogen loss through denitrification. This module tracks the nutrient flow in the nitrogen cycle through mineralization, immobilization, nitrification, denitrification, and urea hydrolysis processes. Simulations involving CRF were conducted by adjusting the urea hydrolysis rate to approximate the CRF conditions. The APSIM model accommodates various fertilizer types, including urea and CRF, by introducing different release periods into the inorganic fertilizer module. The APSIM sunflower module simulated the daily root system growth from germination to grain-filling onset. The increase in root depth was calculated based on the daily growth rate and multiple factors. Daily root biomass growth was proportional to the shoot yield. Each growth stage had a specified root-to-shoot ratio that changed continuously from emergence to flowering. APSIM simulated the daily aging of root mass and length at a specific ratio, with aged roots becoming new organic matter incorporated into the soil nitrogen module.

#### 2.4.3 Data sources and model calibration

This study calibrated the APSIM model using 2020 data and validated it with 2021 data, with the aim of minimizing discrepancies between observed and measured values. APSIM version 7.10 was employed in this study.

In the APSIM model, the calibrated soil parameters are summarized as follows. The soil hydraulic parameters included saturated hydraulic conductivities (*K_s1_*, *K_s2_*, *K_s3_*, and *K_s4_*) with values of 53.1, 32.6, 26.5, and 32.2 mm/mm, respectively, for layers 0–20 cm, 20–40 cm, 40–60 cm and 60–100 cm, respectively. The saturated water contents (*θ_s1_*, *θ_s2_*, *θ_s3_*, and *θ_s4_*) of these layers were calibrated to 0.270, 0.281, 0.442, and 0.462 mm/mm, respectively. The field capacities (*F_c1_*, *F_c2_*, *F_c3_*, and *F_c4_*) were set to 0.243, 0.256, 0.421, and 0.452 mm/mm, respectively, and the wilting point water contents (*θ_wp1_*, *θ_wp2_*, *θ_wp3_*, and *θ_wp4_*) were calibrated to 0.09, 0.09, 0.10, and 0.11 mm/mm, respectively. Nitrogen turnover parameters included a soil nitrification potential of 40 μg NH_4_^-^/g soil, NH_4_^-^ concentration at half potential of 90 ppm, denitrification rate coefficient of 0.0006 kg soil/mg C per day, and a power term (water factor for denitrification) set at 1.

The calibrated genetic coefficient values of the APSIM model for sunflowers are detailed as follows. The effective accumulated temperature from the end of the seedling stage to flower bud differentiation (tt_endjuv_to_init) was calibrated to 450°C/d. The temperature required for flower bud differentiation into flag leaves (tt_fi_to_flag) was set at 430°C/d. The effective accumulated temperature from flowering to the start of grain (tt_flower_to_start_grain) was 150°C/d, and from flowering to maturity (tt_flower_to_maturity) it is calibrated at 1100°C/d. The total leaf area coefficient (tpla_prod_coef) was set at 0.017, and the leaf emergence rate (rel_leaf_init_rate) at 0.5 leaf/d. The leaf senescence coefficient (spla_prod_coef) and the intercept (spla_intercept) were calibrated to 0.0035 and 0.01, respectively. The radiation use efficiency (RUE) is set at 1.15 g/MJ, and the daily increase in the harvest index (hi_incr) was 0.011.

### 2.5 Calculations analysis

(1) Principle and Improvement of the Jaccard Index

In bioinformatics and statistics, the Jaccard index can be widely applied to quantify similarity or overlap between two sample sets (Equation 1) (Real and Vargas, 1996). It is calculated as the ratio of the size of the intersection to the union of two sets, A and B:


(1) $$J(A,B)=\left|\frac{A\cap B}{A\cup B}\right|$$

where A∩B is the number of elements in the intersection of sets A and B, and A∪B is the number of elements in their union.

To apply this index to analyze nitrate-nitrogen content and root length density, these metrics should first be categorized. The nitrate nitrogen content was divided into ten gradations within the 0–48 mg/kg range, with each gradation spanning 4.8 mg/kg. Similarly, the root length density was divided into ten gradations within the 0–2.8 cm/cm^3^ range, with each gradation spanning 0.28 cm/cm³. This categorization assigns each sample point to a specific gradation, thereby simplifying the matching analysis.

This study introduced a weighted mechanism to consider the correlation between adjacent gradations. Even if the samples were not in the same gradation, a certain degree of match was assigned if they were in adjacent gradations, based on their distance. The weight for a perfect match (same gradation) was 1; for adjacent gradations, it was 0.8; and it decreased by 0.2 for more distant gradations, until it reached 0. This weighting reflects the actual differences between the samples, thus enhancing the analysis. The improved formula for the weight calculation is:

The weight calculation formula can be represented as


(2) W(g)=max(0,1−α×|G−g|)

where *W(g)* is the weight at grade g; *G* is the specified grade (e.g., grade of soil nitrate concentration or root length density); g represents the current grade under consideration, ranging from 1 to 10; and *α* is the weight interval, which is a fixed constant that controls the rate of change in weight with distance. In this study, α was set to 0.2.

The weighted intersection and union are calculated as follows:


(3) $$N\cap R=\sum_{g=1}^{10}\min(W_{N}(g),W_{R}(g))$$

(4) $$N\cup R=\sum_{g=1}^{10}\max(W_{N}(g),W_{R}(g))$$

where N∩R is the weighted intersection of soil nitrate nitrogen and root length density, and N∪R is the weighted union, with *W_N_(g)* and *W_R_(g)* being the weights for soil nitrate nitrogen and root length density at gradation g, respectively.

The Weighted Root Jaccard Index is calculated as:


(5) $$RJ_{index}=\frac{\sum_{i=1}^{n}\frac{N\cap R}{N\cup R}}{n}$$

where *RJ_Index_* is the Weighted Root Jaccard Index, and *n* is the number of days per growth stage.

This improved method involves the computation of weights, weighted intersection and union, and Weighted *RJ_Index_*. This approach facilitated a more precise assessment of the match between soil nitrate-nitrogen content and root length density, particularly concerning subtle differences between the soil layers. This advancement provided a new tool for agricultural and ecological research, improving our understanding of the interactions between crop root systems and soil nutrients.

(2) NUE is a key agricultural indicator that measures the efficiency of crop nitrogen fertilizer utilization. It is calculated as the ratio of the crop yield to the amount of nitrogen absorbed by the crop.


(6) $$NUE=\frac{Y}{SNU}$$

where Y (kg/ha) is the yield of each treatment, and SNU (kg/ha) is the sunflower nitrogen uptake.

### 2.6 Model evaluation statistics

In this study, the performance of the model in simulating soil NO_3_-N concentration (SNC), sunflower root length density (RLD), crop nitrogen uptake, and yield was assessed using several statistical methods. These included the Coefficient of Determination (*R^2^*), where a higher value indicated a better fit of the model to the data; Root Mean Square Error (*RMSE*), where lower values indicated a better fit; and Mean Absolute Error (*MAE*), where a lower value indicated more accurate model predictions.


(7) $$R^{2}=1-\frac{\sum_{i=1}^{n}{\left(M_{i}-S_{i}\right)}^{2}}{\sum_{i=1}^{n}{\left(M_{i}-M\right)}^{2}}$$

(8) $$RMSE=\sqrt{\frac{\sum_{i=1}^{n}{\left(M_{i}-S_{i}\right)}^{2}}{n}}$$

(9) $$MAE=\frac{1}{n}\sum_{i=1}^{n}\left|M_{i}-S_{i}\right|$$

Where, *M_i_* represents the observed value, *S_i_* represents the simulated value, *M* is the average of the observed value, and *n* is the sample size.

### 2.7 Statistical analysis

In this study, data processing was conducted using Microsoft Excel 2019, and graphical representations were generated using Origin 2021 software, Python programming was used to calculate the correlation between soil nitrate nitrogen and sunflower root length density.

## 3 Results

### 3.1 Model calibration and validation

**Figure 2:**
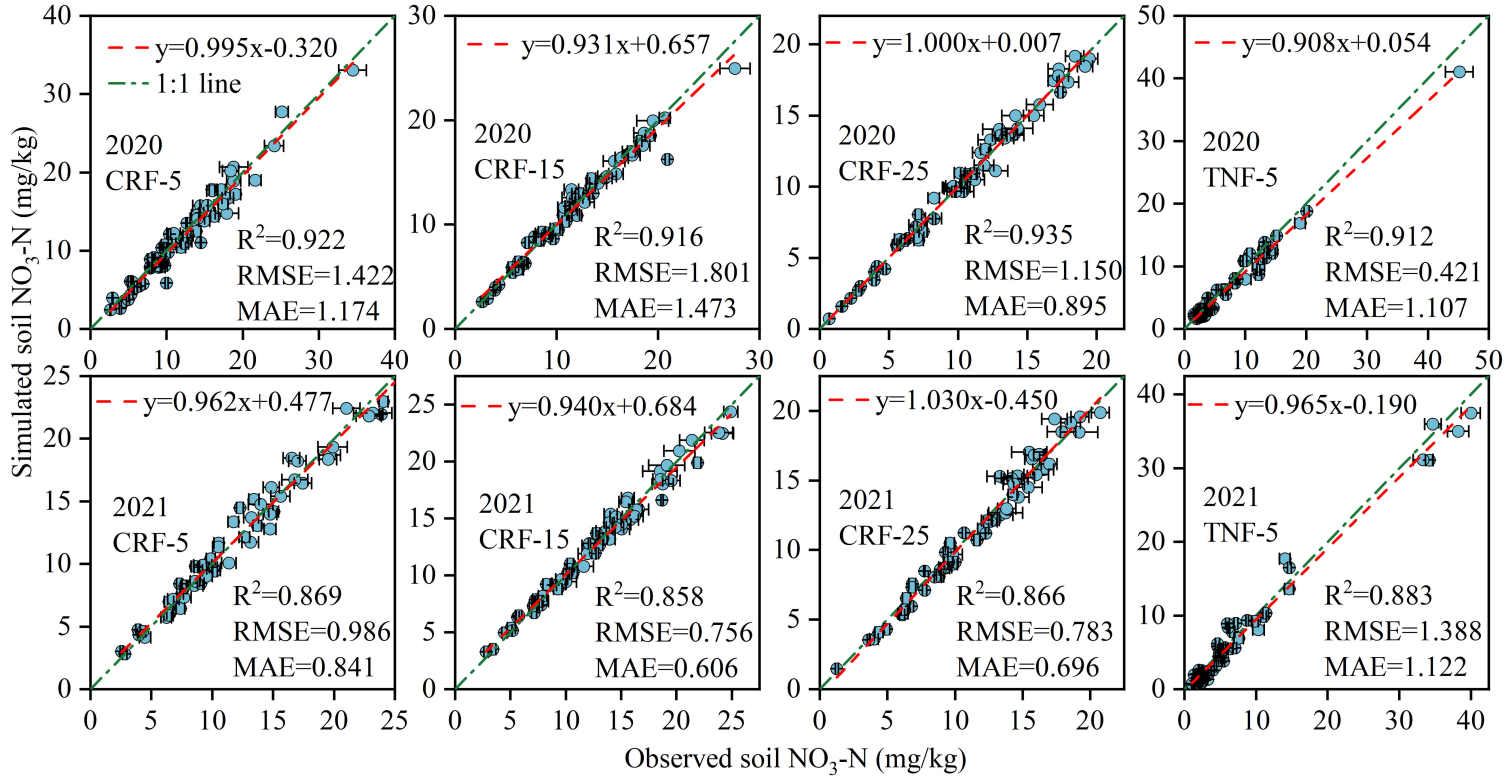
Statistical results for APSIM calibration (2020) and validation (2021) for soil NO_3_-N concentrations (SNC), controlled-release fertilizer (CRF) at three depths of N-fertilizer application (5, 15, and 25 cm) and traditional nitrogen fertilizer (TNF) applied at a 5 cm depth. *R^2^*, *RMSE*, and *MAE* are the determination coefficient, root mean square error, and mean absolute error, respectively.

**Figure 3:**
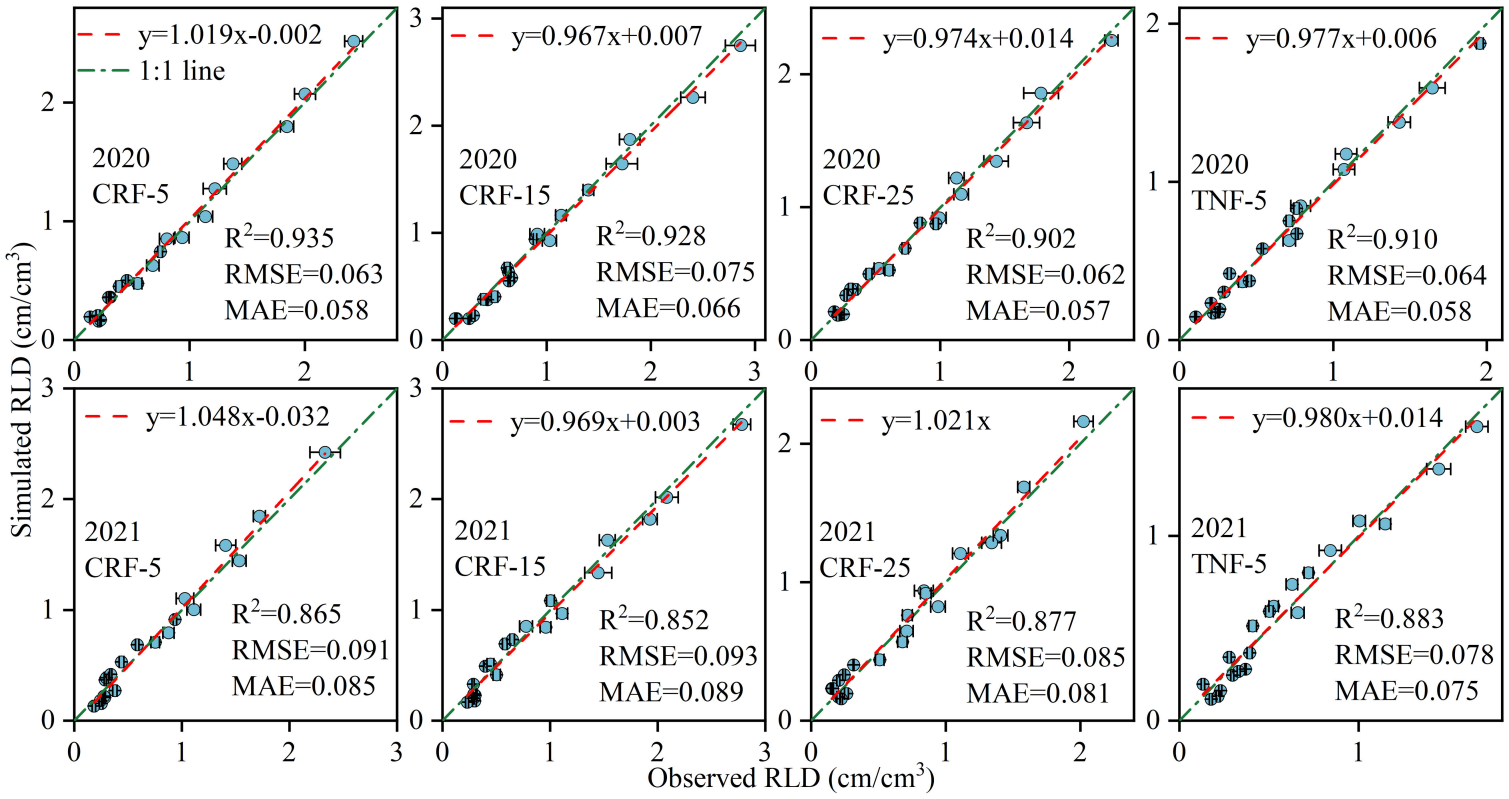
Statistical results for APSIM calibration (2020) and validation (2021) for root length density (RLD), controlled-release fertilizer (CRF) at three depths of N-fertilizer application (5, 15, and 25 cm) and traditional nitrogen fertilizer (TNF) applied at a 5 cm depth. *R^2^*, *RMSE*, and *MAE* are the determination coefficient, root mean square error, and mean absolute error, respectively.

This APSIM model was employed to simulate soil NO_3_-N concentration (SNC), sunflower root length density (RLD), crop nitrogen uptake, and yield throughout the entire growth cycle. The model was calibrated using experimental data from 2020 and validated using data from 2021. Performance evaluation included key indicators such as the coefficient of determination (*R^2^*) (Equation 7), root mean square error (*RMSE*) (Equation 8), and mean absolute error (*MAE*) (Equation 9) (**Figures 2**, **3** and **Table 1**).

**Table 1:** Statistical results for APSIM calibration (2020) and validation (2021) for nitrogen uptake and yield, controlled-release fertilizer (CRF) at three depths of N-fertilizer application (5, 15, and 25 cm) and traditional nitrogen fertilizer (TNF) applied at a 5 cm depth.

Parameter	2020 for Calibration			2021 for Verification		
	*R^2^*	*RMSE* (kg/ha)	*MAE* (kg/ha)	*R^2^*	*RMSE* (kg/ha)	*MAE* (kg/ha)
Yield(kg/ha)	0.891	168.37	157.80	0.895	178.52	145.10
Nitrogen uptake(kg/ha)	0.794	5.07	4.11	0.733	8.88	8.63

*R^2^*, *RMSE*, and *MAE* are the determination coefficient, root mean square error, and mean absolute error, respectively.

During 2020–2021, the *RMSE* values were 0.421–1.801 mg/kg for SNC. 0.062–0.093 cm/cm^3^ for sunflower RLD, 5.07–8.88 kg/ha for sunflower nitrogen uptake, and 168.37–178.52 kg/ha for yield. The average *R^2^* values were 0.895 (SNC), 0.894 (RLD), 0.764 (NU), and 0.893 (yield), with corresponding average *MAE* values of 0.989 mg/kg, 0.071 cm/cm^3^, 6.37 kg/ha, and 151.45 kg/ha. These findings indicate the high precision and strong predictive capability of the model for simulating these variables from 2020 to 2021.

### 3.2 Effects of fertilizer application depth on SNC distribution

**Figure 4:**
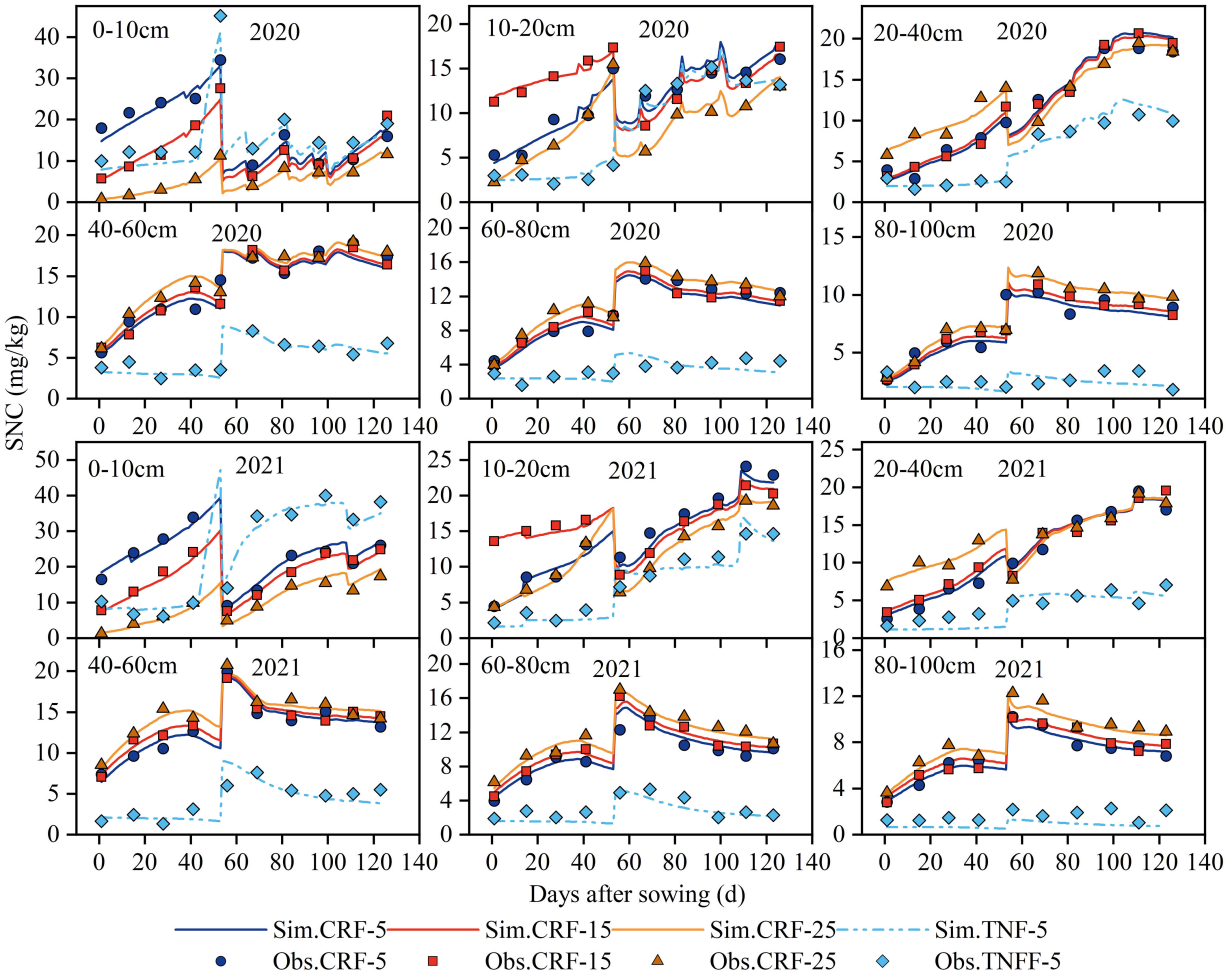
Soil NO_3_-N concentrations (SNC) in 0–100 cm soil under controlled-release fertilizer (CRF) at three depths of N-fertilizer application (5, 15, and 25 cm) and traditional nitrogen fertilizer (TNF) applied at a 5 cm depth from 2020 to 2021.

This study analyzed the 2020 and 2021 experimental data to investigate the relationship between fertilizer application depth and the distribution of SNC (**Figure 4**). The results indicated that at a depth of 5 cm, the SNC in the soil surface was significantly higher than that in deeper layers. In contrast, depths of 15 and 25 cm resulted in higher SNC in the deeper soil layers. Throughout the growth period, SNC under TNF treatment was lower than that under CRF treatment with the same amount of nitrogen. Specifically, during the 2020 and 2021 experimental periods, in the 0–10 cm soil layer during the sunflower seedling stage, the SNC of CRF-5 was 86.83% and 695.73% higher than that of the CRF-15 and CRF-25 treatments, respectively. At the budding and flowering stages, it was higher by 35.73% and 206.27%, respectively. In the 10–20 cm soil layer, at the seedling stage, the SNC of the CRF-15 treatment was 93.63% and 144.94% higher than that of the CRF-5 and CRF-25 treatments, respectively. At the budding and flowering stages, they were 11.08% and 33.28% higher, respectively. In the deep soil layer (20–100 cm), from the seedling stage to the end of the flowering stage, the SNC of the CRF-25 treatment was 19.91% and 13.44% higher than that of the CRF-5 and CRF-15 treatments, respectively. At the maturity stage, within the 0-40 cm soil layer, the SNC of the CRF-5 treatment increased by 5.00% and 18.81% compared with CRF-15 and CRF-25, respectively. In the 40–60 cm soil layer, CRF-25 increased by 11.84% and 8.13% compared with CRF-5 and CRF-15, respectively.

Throughout the growth period, the SNC of the CRF-5 treatment in the 0–100 cm soil layer was 114.01% higher than that of the TNF-5 treatment at the same nitrogen application rate. These findings are important for understanding the effects of deep CRF application on SNC distribution and its potential effects on soil fertility and plant growth.

### 3.3 Effects of fertilizer application depth on sunflower RLD across growth stages

**Figure 5:**
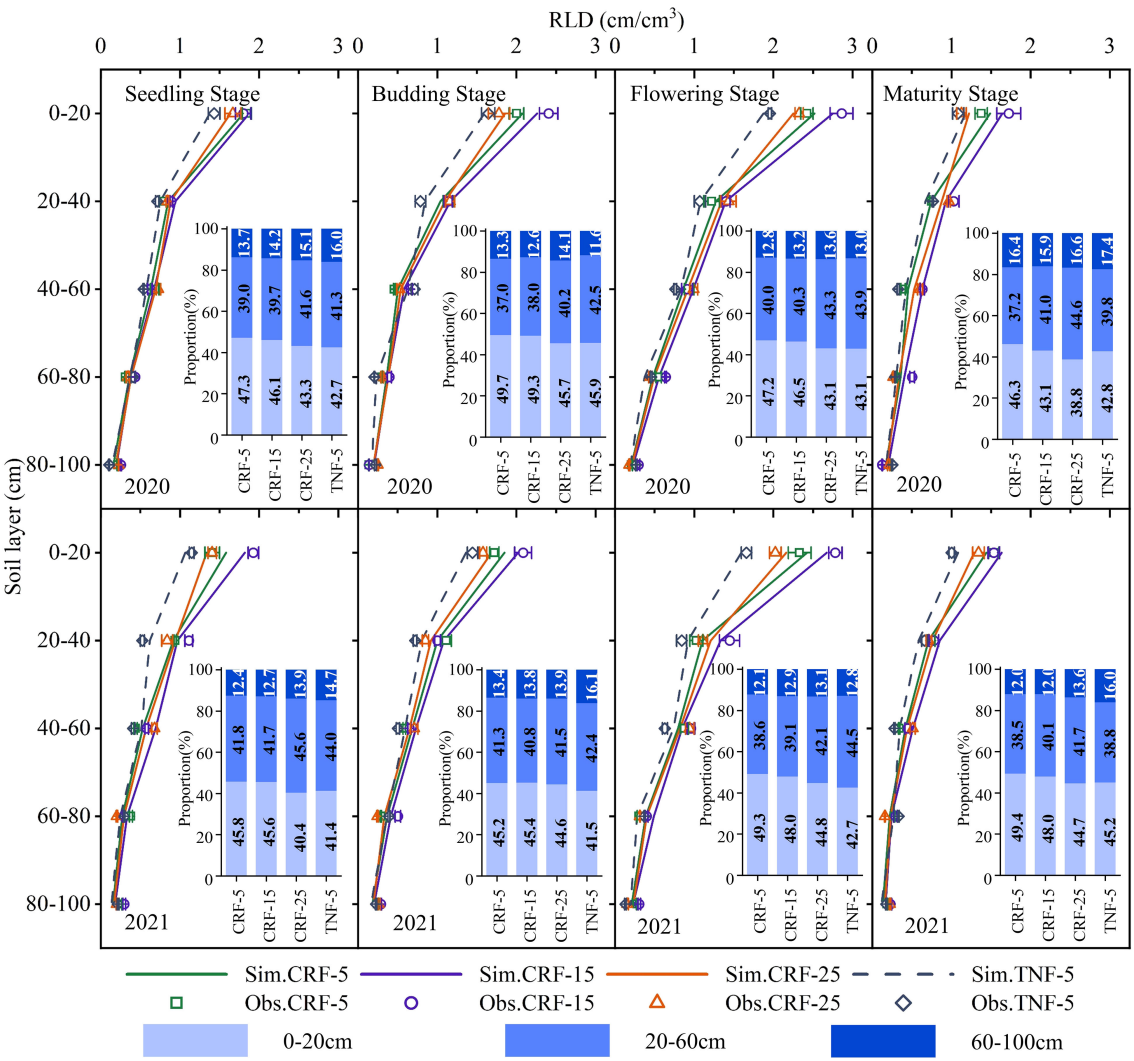
Effects of controlled-release fertilizer (CRF) at three depths of N-fertilizer application (5, 15, and 25 cm) and traditional nitrogen fertilizer (TNF) applied at a depth of 5 cm on the vertical distribution and distribution proportion of different soil layers (bar charts embedded in each figure) of root length density (RLD) of sunflower at seedling, budding, flowering, and maturity growth stages from 2020 to 2021.

The 2020–2021 data demonstrated that within each treatment group, sunflower RLD initially increased and then decreased throughout the growth period. Overall, RLD decreased gradually with increasing soil depth across the entire soil profile (**Figure 5**). Under the CRF-5 treatment, RLD was mainly concentrated in the surface layer, whereas under the CRF-15 and CRF-25 treatments, it increased at greater depths. Specifically, during the seedling stage, the RLD of the CRF-15 treatment in the 0–100 cm soil profile was 11.15% and 14.02% higher than that of the CRF-5 and CRF-25 treatments, respectively. At the budding, flowering, and maturity stages, these differences changed to 9.38% and 15.21%, 12.05% and 14.37%, and 17.63% and 19.67%, respectively, indicating that the RLD under CRF-15 treatment reached its maximum value at all growth stages. These findings suggest that fertilizer depth can influence the downward growth and distribution of the root system and that an appropriate depth can enhance sunflower RLD at different growth stages.

During the seedling, budding, flowering, and maturity stages of sunflower growth, the RLD of the CRF-5 treatment was 26.01%, 22.17%, 27.23%, and 19.32% higher than that of the TNF-5 treatment with the same amount of nitrogen, respectively. This suggests that CRF may enhance the growth of the crop root system by adjusting SNC. The bar charts in each figure illustrate that, under CRF treatments, as the depth of fertilizer application increased, the proportion of RLD in the 0–20 cm soil layer gradually decreased, whereas RLD in the 20–100 cm soil layer showed an increasing trend. These findings highlight the potential of different CRF fertilizer placement positions to optimize crop root distribution and promote downward root growth.

### 3.4 Evaluating the effects of fertilizer application depth using the root system Jaccard index

**Figure 6:**
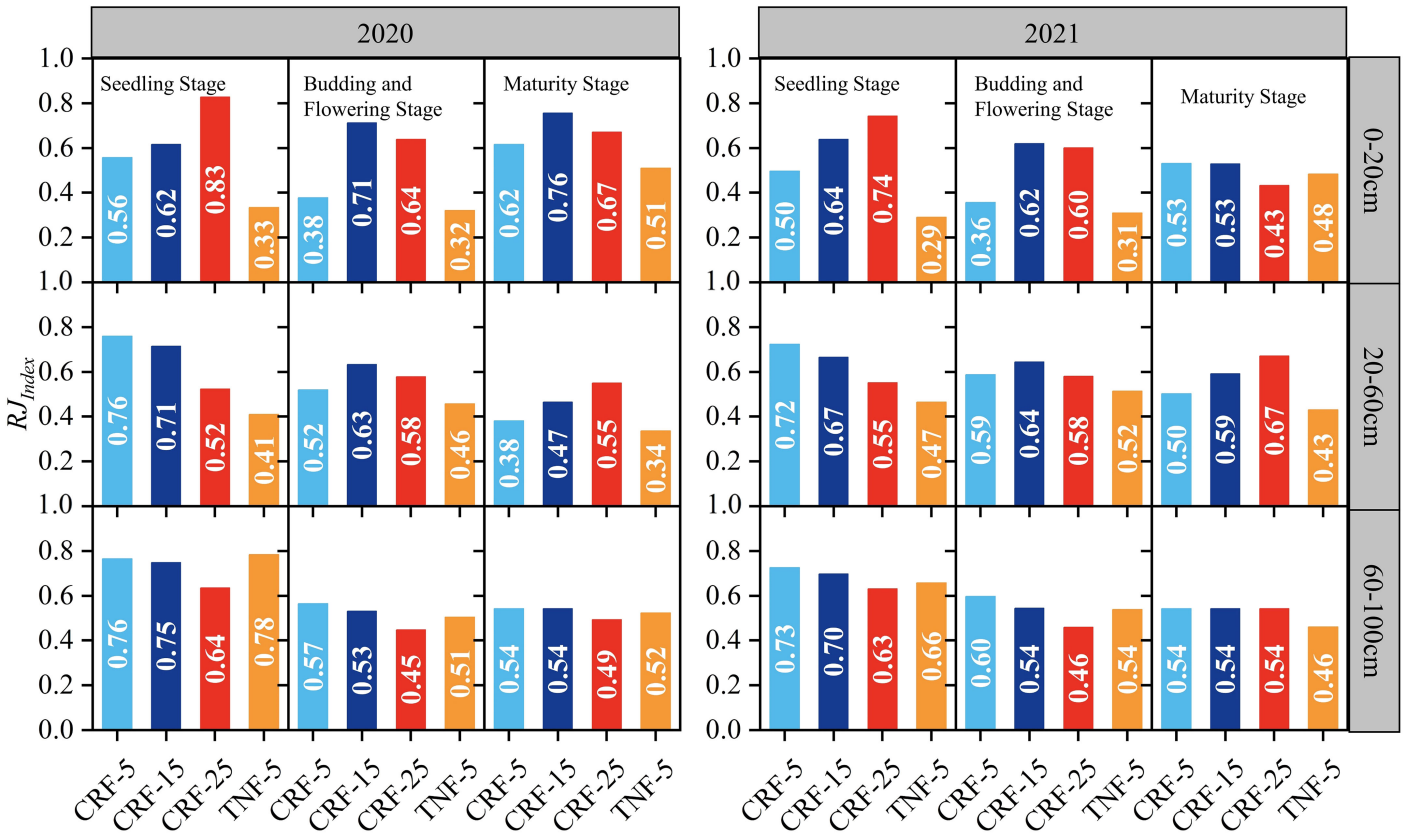
Root Jaccard Index of soil NO_3_-N concentrations (SNC) and root length density (RLD) in the 0–100cm soil profile during the growing season under controlled-release fertilizer (CRF) at three depths of N-fertilizer applications (5, 15, and 25 cm) and traditional nitrogen fertilizer (TNF) applied at a 5 cm depth, from 2020 to 2021.

Using the root system Jaccard index (Equations 2–5) to quantify the match between crop RLD and SNC revealed the effect of CRF treatment depth on their compatibility (**Figure 6**). During the seedling stages of 2020 and 2021, the average Jaccard index of the root system in the 0–20 cm soil layer for the CRF-25 treatment was 48.48% and 34.44% higher than that for the CRF-5 and CRF-15 treatments, respectively, indicating better compatibility between RLD and SNC in the soil surface layer. However, during the budding, flowering, and maturity stages, the CRF-15 treatment had the highest average Jaccard index of root system. Specifically, during the budding and flowering stages, the CRF-15 treatment was 81.28% and 7.27% higher than that of the CRF-5 and CRF-25 treatments, respectively, whereas at the maturity stage, it increased by 11.18% and 17.42%, respectively. In the 20–60 cm soil layer, the Jaccard index ranking of the average root system during the seedling stage was CRF-5>CRF-25>CRF-15. During the budding and flowering stages, it was CRF-15>CRF-5>CRF-25, with the CRF-15 treatment being 15.76% and 10.37% higher than that of the CRF-5 and CRF-25 treatments, respectively. By the maturity stage, it changed to CRF-25>CRF-15>CRF-5, with the CRF-25 treatment being 38.58% and 15.83% higher than the CRF-5 and CRF-15 treatments, respectively. In the deeper 60–100 cm soil layer, the Jaccard index ranking of the root system during the seedling and budding plus flowering stages was CRF-5>CRF-15>CRF-25. At the maturity stage, there was essentially no difference in the average Jaccard index of the root system among the different fertilizer depths.

Throughout the 0–100 cm soil profile, the average Jaccard index of the root system was consistently better for the CRF-15 treatment than for CRF-25 and CRF-5, increasing by 6.60% and 7.34%, respectively, during the seedling, budding plus flowering, and maturity stages. This suggests that CRF application at a depth of 15 cm can significantly improve the match between RLD and SNC throughout the growth period. Additionally, the root system Jaccard index for the CRF-5 treatment was 20.45% higher than that for the TNF-5 treatment with the same nitrogen application rate, indicating that CRF treatments can better optimize the match between RLD and SNC than TNF treatments.

### 3.5 Effects of fertilizer application depth on sunflower yield, nitrogen uptake, and NUE

This study investigated the effects of different CRF fertilizer application depths (5, 15, and 25 cm) on sunflower yield, nitrogen uptake (NU), and NUE in field experiments (**Table 2**). Synthesizing the data from 2020 and 2021 revealed a significant impact of fertilizer application depth on these indicators. In terms of yield, the CRF-15 treatment outperformed the other treatments in both years, with yields of 8.62% and 10.25% higher than the CRF-5 and CRF-25, respectively, indicating that medium-depth fertilization promoted crop growth and yield. Compared with TNF-5, the CRF-5 increased the yield by 30.62% under the same nitrogen application rate. CRF-15 exhibited the highest NU capacity, which was 4.82% and 5.97% higher than that of CRF-5 and CRF-25, respectively, highlighting the role of medium-depth fertilization in promoting effective NU. Regarding NUE (Equation 6), CRF-15 also exhibited higher efficiency, with NUE 3.63% and 4.04% higher than those of CRF-5 and CRF-25, respectively. This suggests that at a depth of 15 cm, the NUE was optimized, enhancing the nitrogen absorption and utilization efficiency of the crop. Compared to TNF-5, under the same nitrogen application rate, the NU and NUE under CRF-5 increased by 14.95% and 13.45%, respectively, indicating that CRF treatment significantly improved yield, NU, and NUE compared to TNF.

**Table 2:** Biennial comparative analysis of yield, nitrogen uptake (NU), and nitrogen use efficiency (NUE) under controlled-release fertilizer (CRF) at three depths of N-fertilizer application (5, 15, and 25 cm) and traditional nitrogen fertilizer (TNF) applied at a 5 cm depth from 2020 to 2021.

Years	Treatment	Sim.yield	Obs.yield	Sim.NU	Obs.NU	NUE
		kg/ha	kg/ha	kg/ha	kg/ha	kg/kg
2020	CRF-5	3886.00	4092.81 ± 56.09ab	236.7	238.70 ± 4.54ab	16.42
	CRF-15	4239.30	4385.20 ± 259.39a	246.4	249.40 ± 9.27a	17.20
	CRF-25	3789.70	3723.10 ± 92.63b	223.8	221.60 ± 7.87b	16.93
	TNF-5	3231.00	3019.11 ± 78.53c	215.5	224.72 ± 2.47b	14.99
2021	CRF-5	4246.30	4115.60 ± 98.77ab	241.7	248.60 ± 10.07a	17.57
	CRF-15	4471.90	4415.00 ± 118.55a	252.1	260.10 ± 0.45a	17.73
	CRF-25	4178.10	3859.20 ± 227.56b	247.6	240.20 ± 3.78a	16.87
	TNF-5	3012.50	2938.60 ± 98.48c	201.3	213.50 ± 12.21b	14.97

Lowercase letters (e.g., a, b, c, d) are used to indicate significant differences between groups. The significance level is determined based on statistical tests and is set at p < 0.05.

Overall, these findings suggested that CRF application at a depth of 15 cm significantly enhances sunflower yield, NU capability, and NUE. This depth likely benefits from being closer to the main root zone of the crop, facilitating effective nitrogen absorption and utilization by the root system.

## 4 Discussion

### 4.1 Comprehensive analysis of influencing factors

#### 4.1.1 Impact of fertilization depth on SNC distribution

This study indicated a significant impact of CRF application depth on SNC distribution, which is crucial for enhancing NUE and reducing environmental pollution (Xia et al., 2022). Shallow CRF application (5 cm) resulted in notably higher NO_3_-N concentrations in the surface soil than in the deeper layers, whereas deeper application (15 and 25 cm) increased NO_3_-N concentration in the lower soil layers (**Figure 4**). This is consistent with previous findings (Wu et al., 2022a; Chen et al., 2022c). The impact of CRF application depth on SNC distribution was multifaceted. Shallow fertilization leads to surface soil NO_3_-N accumulation owing to higher evaporation rates and limited water penetration, whereas deep fertilization promotes the downward NO_3_-N percolation with soil moisture, reaching deeper layers (Kanwar et al., 1985). Moreover, fertilization depth may influence root system efficiency in NO_3_-N absorption. The shallow fertilization can be more readily absorbed, affecting NO_3_-N distribution, whereas deep fertilization may stimulate downward root growth for NO_3_-N absorption from the deeper soil layers (Liu et al., 2018).

In summary, the effect of fertilization depth on SNC can be influenced by various factors, such as moisture dynamics, microbial activity, and root absorption. Understanding these mechanisms is crucial for guiding fertilization strategies in modern agriculture with the aim of enhancing nitrogen fertilizer efficiency and mitigating environmental pollution.

#### 4.1.2 Correlation between RLD and SNC

RLD is a crucial indicator of root distribution in the soil, directly affecting the ability of a crop to absorb NO_3_-N (Dusserre et al., 2009). This study demonstrated a strong correlation between RLD and SNC, highlighting the close link between soil nitrogen availability and crop root system structure. Across different fertilizer depth conditions, changes in root length density reflect the adaptation of the root system to nitrogen availability (Liu et al., 2018; Chen et al., 2022a). Shallow fertilization (5 cm) resulted in a higher RLD in the surface soil, indicating root growth in areas with higher SNC (Jia et al., 2020). In contrast, deep fertilization (15 and 25 cm) led to relatively higher RLD in deeper soil layers, suggesting downward root growth to access nitrogen in deeper layers (**Figure 5**) (van der Bom et al., 2020).

Root development and distribution are vital mechanisms for crops to adapt to the soil environment, particularly regarding soil nutrient distribution (Giehl and von Wiren, 2014; Zhao et al., 2020; Kang et al., 2022). The RLD distribution indicated that crop roots could adjust their growth based on the NO_3_-N distribution, thereby optimizing nutrient absorption (Chen et al., 2020). To effectively absorb the nitrogen, roots should maintain an appropriate growth density in high-N areas (Fageria and Moreira, 2011). Therefore, adjusting fertilizer depth to optimize root growth and the distribution is a potential strategy for influencing soil nitrogen distribution. For instance, medium-depth CRF fertilization (15 cm) may better match the fertilizer with the main root system distribution area, improving nitrate-nitrogen absorption efficiency (**Figure 6**).

In summary, the correlation between root length density and SNC demonstrated how crop roots responded and adapted to soil nitrogen distribution. This understanding is vital for developing effective fertilization strategies, particularly for enhancing NUE and crop growth. Adjusting fertilizer depth can optimize SNC distribution and influence root structure and function, thereby improving nitrogen absorption and utilization efficiency (Morris et al., 2017).

### 4.2 Impact of fertilization depth on crop yield, nitrogen absorption, and strategies to improve NUE

In this study, different CRF fertilization depths (5, 15, and 25 cm) exhibited significant effects on the sunflower yield, NU, and NUE (**Table 2**). These effects could result from two main factors. First, fertilization depth directly affected NO_3_-N distribution in the soil. Second, it indirectly affected root system distribution and functionality. NO_3_-N distribution is critical for crop growth and yield, as higher concentrations in areas with denser root systems lead to increased absorption efficiency, which is conducive to higher yields (Barber and Mackay, 1986). Therefore, alignment between fertilization depth and root system distribution is crucial for maximizing yield.

In a spring maize experiment (Wu et al., 2021), trials were conducted at fertilizer depths of 5, 15, 25, and 35 cm. The results indicated that at 15 cm and 25 cm depths, maize yield increased by 5.68% and 13.83%, respectively, compared to the 5 cm depth, with NUE improving by 17.79% and 38.37%, respectively. The highest yield and NUE were observed at a depth of 25 cm. However, the 15 cm depth treatment yielded 3.90% and 4.32% higher NUE than the 5 cm and 25 cm treatments, respectively, with average NUE increases of 0.67% and 1.56%, respectively. The highest yield and NUE were obtained at the depth of 15 cm (**Table 2**). These variations in results may be attributed to meteorological conditions such as precipitation, soil texture, and initial nutrient concentration in the soil. Overall, the study suggested that appropriately increasing CRF application depth can enhance the crop yield and NUE in agricultural production. Medium-depth fertilization may be preferred because it avoids the excessive SNC in the surface layer and reduces the NO_3_-N distribution in deeper soil layers, ensuring more even nitrogen distribution across soil layers where crop roots are concentrated. This depth also minimizes N volatilization and leaching losses (Min et al., 2021; Wu et al., 2021), enhancing the availability of NO_3_-N for crop absorption and use. In medium-depth soils, N had a longer contact time with the root systems, improving NUE. Furthermore, the soil moisture and temperature conditions at this depth may promote root growth and enhance microbial activity, which facilitates N transformation through mineralization, making it more readily absorbable by crops (Niu et al., 2021). Therefore, this study demonstrated that the medium-depth application of CRF (such as at 15 cm) performed best, likely due to the creation of favorable conditions for crop nitrogen absorption, resulting in improved yield and NUE (Chen et al., 2022b). These findings are significant for guiding agricultural practices, particularly the application of precision fertilization techniques, to enhance NUE and crop productivity.

### 4.3 Future research directions and practical applications

The findings of this study hold practical value in agriculture. Optimizing CRF application depth can improve NUE, reduce costs (Sainju et al., 2006), and increase yields (Liu et al., 2022; Wu et al., 2022b), thus benefiting small-scale farmers with limited resources. This optimization also mitigates the environmental impact of nitrogen fertilizers, which could be crucial for addressing climate change and protecting ecosystems (Wu et al., 2021). The application of the weighted Jaccard index proposed a refined method for analyzing soil-root system interactions. This index considered not only the direct match between the SNC and RLD but also the match between adjacent gradations. While showing advantages, the practical application of the weighted Jaccard index faced challenges such as selecting the appropriate gradation and weight distribution, which significantly affected the analysis results. Future research should focus on effectively determining these parameters effectively to ensure the accuracy and reliability of the analysis. Additionally, further exploration is needed regarding the performance of the index in handling extreme values. Future studies could also investigate its application in broader agricultural and ecological research, such as assessing the responses of different crop varieties to soil nutrients or evaluating the impact of climate change on soil-root system interactions (Cangioli et al., 2022; Lamichhane et al., 2024).

Future research should be conducted under a broader range of geographical and climatic conditions to verify the general applicability of the findings of this study, considering the influence of different soil types and environmental conditions. It is also crucial to investigate how different crop varieties respond to fertilization depth, and how this interacts with crop genetic and physiological traits. Additionally, long-term experimental studies are needed to understand the lasting impact of fertilization depth on soil health and ecosystem services, such as carbon storage and biodiversity.

## 5 Conclusion

The APSIM model was used to analyze the effects of CRF application depth on SNC, sunflower RLD, nitrogen uptake, and yield. The results showed that CRF depth significantly influenced SNC distribution, affecting root growth and nitrogen absorption. Shallow fertilization increased the NO_3_-N concentration in the soil surface layer, whereas deep fertilization moved NO_3_-N to deeper soil layers. Medium-depth fertilization at 15 cm indicated the best performance in enhancing sunflower yield and nitrogen absorption, highlighting the importance of optimizing CRF application depth to improve NUE and promote crop growth. The application of an improved Jaccard index provided a new method for quantifying soil-root system interactions and enhancing understanding. However, the applicability of this study was limited to specific environmental and crop conditions. Future studies should validate these findings across a broader range of environmental and crop varieties. Further exploration of the long-term impacts of adjusting the fertilization depth on soil health and ecosystem services is necessary.

## Data Availability

The original contributions presented in the study are included in the article/supplementary material. Further inquiries can be directed to the corresponding author.
